# MMGan: a multimodal MR brain tumor image segmentation method

**DOI:** 10.3389/fnhum.2023.1275795

**Published:** 2023-12-05

**Authors:** Leiyi Gao, Jiao Li, Ruixin Zhang, Hailu Hanna Bekele, Junzhu Wang, Yining Cheng, Hongxia Deng

**Affiliations:** Department of Artificial Intelligence, College of Computer Science and Technology, Taiyuan University of Technology, Taiyuan, China

**Keywords:** image segmentation, brain tumor, multi-modality, pretreatment, depth residual structure, generative adversarial networks

## Abstract

Computer-aided diagnosis has emerged as a rapidly evolving field, garnering increased attention in recent years. At the forefront of this field is the segmentation of lesions in medical images, which is a critical preliminary stage in subsequent treatment procedures. Among the most challenging tasks in medical image analysis is the accurate and automated segmentation of brain tumors in various modalities of brain tumor MRI. In this article, we present a novel end-to-end network architecture called MMGan, which combines the advantages of residual learning and generative adversarial neural networks inspired by classical generative adversarial networks. The segmenter in the MMGan network, which has a U-Net architecture, is constructed using a deep residual network instead of the conventional convolutional neural network. The dataset used for this study is the BRATS dataset from the Brain Tumor Segmentation Challenge at the Medical Image Computing and Computer Assisted Intervention Society. Our proposed method has been extensively tested, and the results indicate that this MMGan framework is more efficient and stable for segmentation tasks. On BRATS 2019, the segmentation algorithm improved accuracy and sensitivity in whole tumor, tumor core, and enhanced tumor segmentation. Particularly noteworthy is the higher dice score of 0.86 achieved by our proposed method in tumor core segmentation, surpassing those of stateof-the-art models. This study improves the accuracy and sensitivity of the tumor segmentation task, which we believe is significant for medical image analysis. And it should be further improved by replacing different loss functions such as cross-entropy loss function and other methods.

## 1 Introduction

Brain tumors are a severe medical condition that can cause significant damage to the nervous system. Among all types of brain tumors, gliomas are known to have the high mortality and morbidity rates (Zhang and Liu, 2022). While the grayscale of tumors and normal brain tissue may be similar, there are significant differences in image intensity, texture, size, shape, location, and other characteristics within the same tumor, making it challenging to obtain comprehensive evaluation information about brain tumors from different modalities of magnetic resonance imaging (MRI) through the eyes of a physician alone (Liang et al., 2021).

Artificial intelligence healthcare refers to the application of computer vision, speech recognition, natural language processing, machine learning, and other artificial intelligence technologies in the medical field. In recent years, with the accelerated maturity of artificial intelligence technology, its application scenarios in the field of healthcare are constantly enriched, bringing profound changes to the mode of disease detection, diagnosis and treatment. Computer-aided diagnosis has gained traction in recent years, making it easier for physicians to qualitatively and quantitatively analyze brain tumors (Mofatteh, 2021). One of the initial steps in this process is the segmentation of lesions in the image, which assists physicians in identifying and analyzing structural, motion, and 3D visualization characteristics of the tumor. This greatly improves the accuracy and reliability of medical diagnosis while providing a better understanding of how tumors affect the brain. Furthermore, computer-aided diagnosis can play an important auxiliary role in medical teaching, surgical planning, surgical simulation and various medical research (Menze et al., 2015; Havaei et al., 2016). Accurate and automatic segmentation of brain tumors in different modalities of brain MRI is a challenging task in medical image analysis (Mecheter et al., 2022).

The convolutional neural network (CNN) algorithm, which integrates three structural ideas: local receptive field, weight sharing, and spatial subsampling, has shown great promise in image segmentation. The CNN algorithm's constant stability of displacement and deformation makes it easier to recognize and extract targets in the image despite displacement, rotation, or scaling (Zhou et al., 2017). The most classical CNN model for brain tumor lesion image segmentation is the 2D-CNN model with dual-path parallel paths proposed by Havaei et al. (2016). This model adopts a cascade structure, which can consider global and local information simultaneously and has better robustness. However, its network segmentation performance is limited because it is only based on a single 2D level and does not consider the correlation of multiple 2D or 3D levels. Later, Shelhamer et al. (2017) introduced fully convolutional networks (FCN), which use a deconvolutional layer instead of the final fully connected layer, so input images of any size can be segmented, achieving pixel-level recognition performance. Sarwar et al. (2017) extended the standard FCN by using a multiscale loss function. Multiscale loss provides different resolutions, and the FCN variant reduces the multiscale loss function by combining higher and lower resolutions to simulate context in the image and label domains. However, the results of FCN's upsampling procedure often need to be more explicit and sensitive to small details of the image, limiting their performance in medical image analysis. U-Net (Ronneberger et al., 2015) is a significant FCN mutant that has been successful in medical image analysis. The U-Net consists of a shrinking path that captures context and a symmetric expanding path that enables precise localization. Isensee et al. (2018) proposed an improved U-Net for brain tumor segmentation, in which they effectively avoided overfitting by employing a dice loss function and extensive data augmentation. In Agarwal et al. (2021), the authors use zero padding to preserve the output dimensionality of all convolutional layers in the downsampling and upsampling paths.

Generative adversarial networks (GANs) have made significant breakthroughs in various fields, including image classification, object detection, and high-resolution image generation (Pradhyumna and Mohana, 2022). GAN-based image segmentation methods have been increasingly used in medical image research because they require fewer training data, generate good effects, and easily integrate with other neural networks (Zhu et al., 2022) in recent years. Therefore, this study will also perform experiments based on GAN and improve the model according to the data characteristics.

The first study used a level set approach to preprocessing multimodal brain tumor data partially. Only 2% of MRI areas were gliomas, which were morphologically diverse and infiltrative, with indistinct borders, and areas of normal brain tissue account for most MRI images. This proportional structure has resulted in severe data imbalance phenomena, which may affect the accuracy of the segmentation algorithm in MRI. A level set method was used to preprocess the data to address these issues. Specifically, the *E*^*CA*^ energy functional of the Canny operator combined with the Local Binary Fitting (LBF) energy functional (Deng et al., 2021) of the traditional level set was used to strengthen the detection of the target edge. Additionally, each modal image was cropped using the convex hull minimum circumscribed matrix algorithm to more accurately remove the redundant background, effectively preventing multisegmentation of the target area, and reducing data imbalance.

The second research content is the extraction of brain tumors using GAN-based methods. A network model was proposed to extract multimodal brain tumors that combine the advantages of deep residual learning units and generative adversarial networks. The general network model may ignore the correlation between image pixels when processing medical images, despite its importance in dealing with details. The parameters of GAN are relatively large, and the structure is only sometimes stable. To solve these problems, a generative semantic segmentation model based on the GAN model is designed using the U-Net network structure and the deep residual in the encoder-decoder structure. The deep residual network learning unit replaces the original convolutional neural network layer, making training of deep networks more convenient. It utilizes identity mapping to facilitate training, effectively reduce the decay of gradient correlations while reducing network parameters and acquiring shallow image features. After the deep residual learning unit in the coding structure, an attention mechanism called SEblock is added to amplify valuable feature channels by changing the channel weights. The decoding part uses skip connections to combine the important low-level detail information and high-level semantic information after SEblock, extracting abstract features through the deep residual learning unit, making the network easy to train while improving the segmentation accuracy, and finally realizing brain tumor segmentation.

## 2 Materials and methods

### 2.1 Datasets

**Figure 1:**
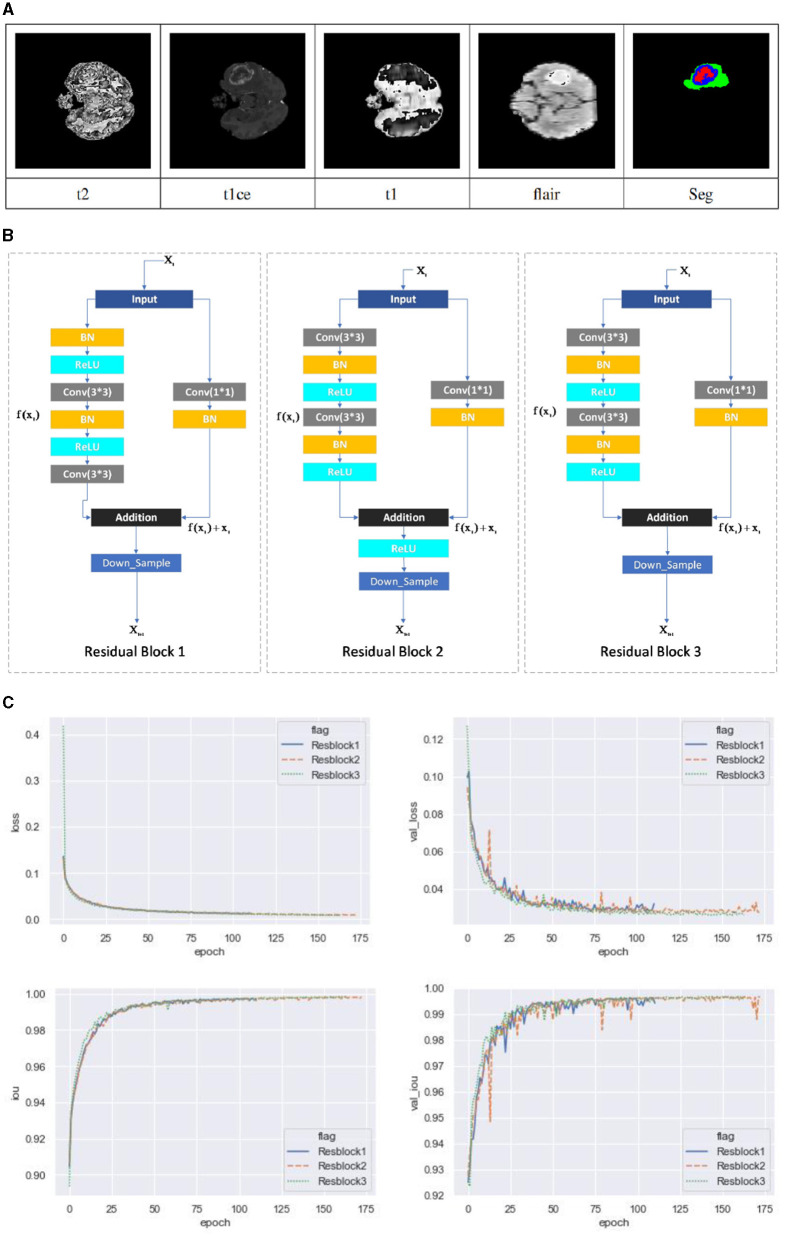
**(A)** BraTS Dataset Display. The MRI images used in this context include T1-weighted MXI (T1), T1-weighted MRJ with gadolinium enhancing contrast (T1c), T2-weighted MXI (T2), and T2-weighted MXI with nuid attenuated in version recovery (FLAIR). Each of these four different MRI modalities can reveal different brain tissues with varying degrees of clarity. **(B)** Residual unit. Three different residual units are designed. Residual Block 1 consists of batch normalization, ReLU activation function, and convolution layer. The arrangement in Residual Block 2 and Residual Block 3 is the convolutional layer, batch normalization and ReLU activation function. The difference is whether an activation function is used before output. **(C)** Loss and iou of different residual structures under different iterations. After the first iteration, Residual Block 1 has the minimum loss value, while Residual Block 2 has the minimum loss value after the tenth iteration. As the number of times increases, the residual unit with the smallest loss is Residual Block 3. This paper adopts the third structure to construct the deep learning network based on the experimental results.

The MRI images used in this context include T1-weighted MXI (T1), T1-weighted MRJ with gadolinium enhancing contrast (T1c), T2-weighted MXI (T2), and T2-weighted MXI with nuid_attenuated in version recovery (FLAIR) as shown in Figure 1A. Each of these four different MRI modalities can reveal different brain tissues with varying degrees of clarity. The Multimodal Brain Tumor Segmentation Challenge (BRATS) was introduced by the Medical Image Computing and Computer Assisted Intervention Society in 2012 to develop and test state-of-the-art brain tumor segmentation algorithms. The challenge involves using an extensive collection of multimodal brain scans that are publicly available in the BraTS database. Each case in the dataset was accurately marked by physicians, reviewed by experienced radiologists, and reviewed by professional physicians.

This paper utilizes the BraTS 2018 and BraTS 2019 datasets, with the former containing a total of 285 cases (210 HGG and 75 LGG), each of which contains four 3D MRI modalities (T1, T2, Flair, T1ce) and a segmented image (seg). The data type is XX.nii.gz, and each modality's data have been resampled to a resolution of 1 × 1 × 1 mm after deskulling. The data size of each modality has been processed to 240 × 240 × 155. Different MRI scanner facilities have collected the data from 19 different institutions.

The BraTS 2019 dataset includes 335 cases (259 HGG and 76 LGG), with 50 additional cases over the BraTS 2018 dataset. The tumor part in these dataset has been divided into three parts according to the doctor's annotation standard: the enhancement tumor area (ET, label = 4, red), the whole tumor area (WT, label = 2, green), and the tumor core area (TC, label = 1, blue).

### 2.2 Related work

#### 2.2.1 Pixel2Pixel

Pixel2Pixel (Isola et al., 2017) is a conditional generative adversarial network. The overall framework of the generator uses U-Net, that is, adding skip connections in the middle of encoding and decoding and copying the underlying information to the high-level feature map. The discriminator uses the auxiliary L1 loss and patchGAN discriminators. The discriminator does not need to see the information of the whole image to make a judgment; instead, they just need to pay attention to the local structure of the image. Compared with the situation where the global image needs to be judged, the number of parameters in training is reduced. The images generated by L1 loss supervision will be more explicit and similar to the ground truth.

#### 2.2.2 Residual network

The introduction of residual networks (He et al., 2016a) marked a significant milestone in the development of deep learning networks. The concept of identity mapping enabled data streams to flow seamlessly across layers, theoretically resulting in better performance. However, deeper networks became more challenging to train and optimize in practice. This is where shortcut connections come in handy. By adding them, residual networks could overcome this problem and simplify the optimization process. A residual block is a few-layer network that incorporates a shortcut connection. It can consist of various combinations of batch normalization (BN), the ReLU activation function, and the convolutional layer. In He et al. (2016b), the authors discuss the impact of different combinations and propose Residual Block 1 shown in Figure 1B, with a fully preactivated design. This work adopts a third residual unit to build the deep residual network, following a comparison between the fully preactivated residual unit and the other two residual structures.

As shown in Figure 1B, three different residual units are designed, with Residual Block 1 being the structure proposed in the paper (Dey and Ashour, 2018). Its arrangement consists of BN, ReLU activation function, and convolution layer. The arrangement in Residual Block 2 and Residual Block 3 is the convolutional layer, batch normalization and ReLU activation function. The difference is whether an activation function is used before output. The three structures are evaluated using the dataset in this paper, and the results are shown in Figure 1C. After the first iteration, Residual Block 1 has the minimum loss value, while Residual Block 2 has the minimum loss value after the tenth iteration. As the number of times increases, the residual unit with the smallest loss is Residual Block 3. This paper adopts the third structure to construct the deep learning network based on the experimental results.

#### 2.2.3 Segmentation methods

Segmentation of tumors from MRI data is a crucial step in diagnosing and treating cancer. Multiple segmentation techniques are available, including but not limited to watersheds, clusters and level sets (Kumar, 2023). Among these, the watershed algorithm is commonly used for the transformation of grayscale images (Khan et al., 2019). The algorithm's primary operational steps include converting the grayscale color images, finding the gradient map, and finally performing the watershed algorithm on the gradient map to obtain the edge line of the segmented image. The K-means clustering algorithm can segment regions of interest from the image background (Khan et al., 2021). The algorithm works by dividing the sample collection into k subsets to form k classes and dividing a given image sample into k classes based on the smallest distance from the point in each class to the center of the class. The level set algorithm is an algorithm used to solve curve evolution problems. The principle algorithm treats a low-dimensional curve as a zero-level set of high-dimensional surfaces and divides the curve through the evolution iteration of the curve. Kaur et al. (2019) utilized various machine learning methods, such as random forest, support vector machine, decision tree and other machine learning methods, to evaluate the performance of tumor segmentation. Similarly, Bansal et al. (2021) used different classification methods to extract target features, including decision trees, KNN, and random forests.

### 2.3 Learning rate

**Figure 2:**
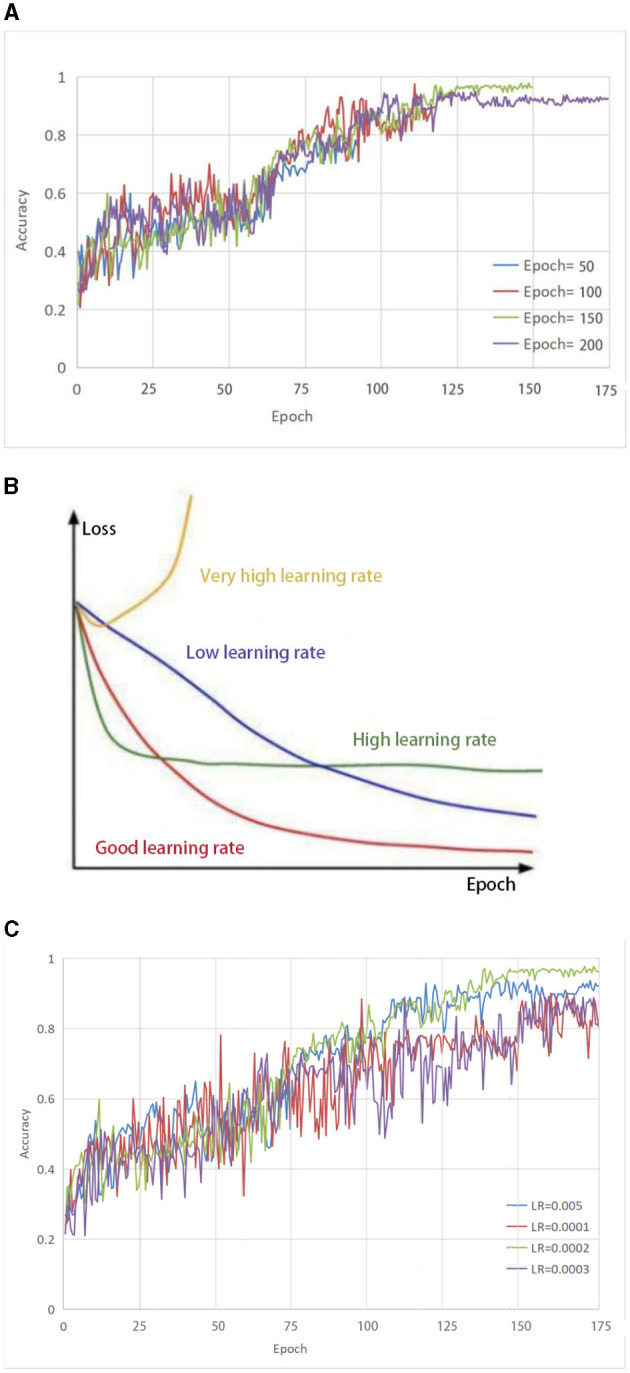
**(A)** Selection of parameter Epoch. The accuracy growth rate is the largest when the number of iterations is 50 and 100, respectively, indicating that the model has not yet reached a converged state. When the number of iterations is set to 150, the highest accuracy can be achieved. As the number of iterations increases, the accuracy remains almost constant. **(B)** The impact of different learning rates on the model. **(C)** Selection of parameter LR. both too-high and too-low learning rates have different effects on the loss of the model. Only a learning rate suitable for our model can make the model converge the fastest and have the least loss in the shortest time.

From Figure 2A, it can be seen that the accuracy growth rate is the largest when the number of iterations is 50 and 100, respectively, indicating that the model has not yet reached a converged state. When the number of iterations is set to 150, the highest accuracy can be achieved. As the number of iterations increases, the accuracy remains almost constant. This indicates that model retraining is no longer valid and therefore the number of iterations was set to 150.

To effectively train the proposed model for medical brain image segmentation, it is essential to find an appropriate learning rate. The learning rate directly affects the convergence of the model, and choosing an optimal learning rate can significantly accelerate the training process. It can be seen from Figure 2B that both too-high and too-low learning rates have different effects on the loss of the model. Only a learning rate suitable for our model can make the model converge the fastest and have the least loss in the shortest time.

To determine the optimal learning rate for our model, we first set the number of iterations to 150 and then performed experiments at different learning rates, including 0.005, 0.0001, 0.0002, and 0.0003. As shown in Figure 2C, the solid green line indicates the highest accuracy when the number of iterations is 150, so the learning rate of this model is set to 0.0002.

### 2.4 Evaluation metrics

The Dice similarity coefficient is a widely used evaluation index for medical image segmentation. The Dice similarity coefficient measures the degree of overlap between the real and predicted regions of an image and is calculated by comparing the overlapping area of the two regions with the total area of the two regions. A Dice score of 1.0 indicates perfect segmentation. The Dice score was calculated using the following formula:


(1) $$\begin{array}{l} Dice=\frac{2TP}{FP+2TP+FN} \end{array}$$

**Figure 3:**
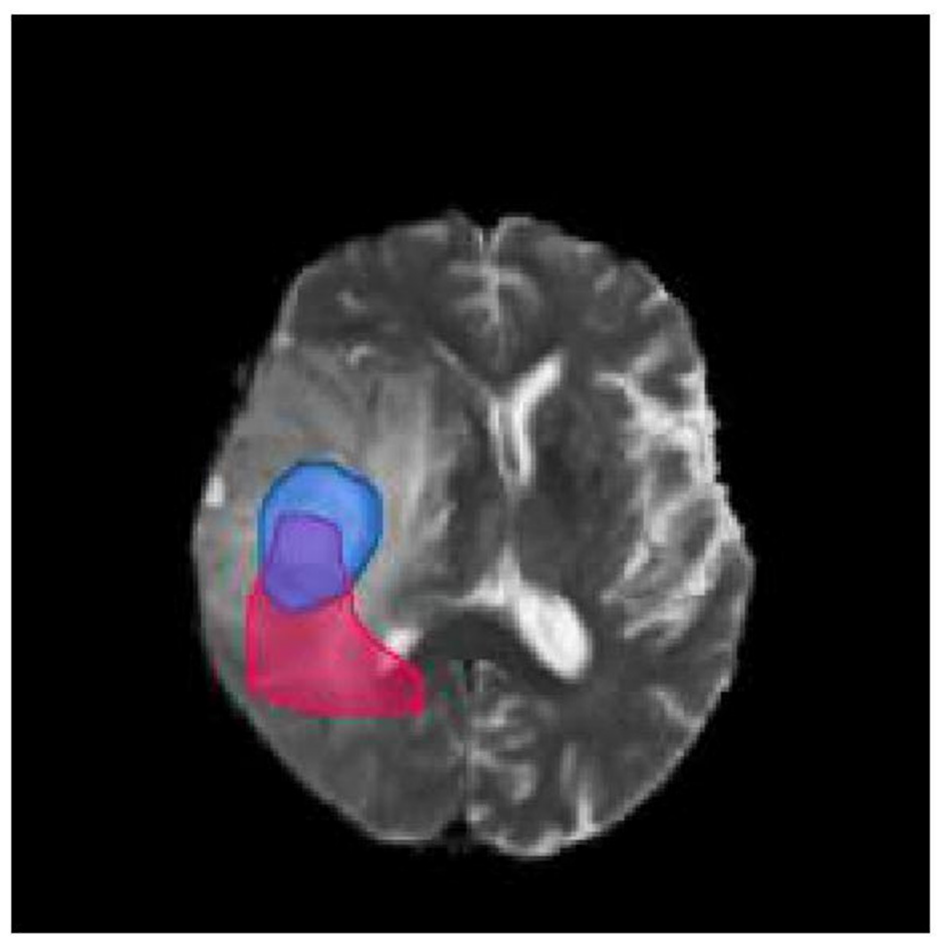
Brain tumor map. The blue part of the image represents the real brain tumor region (ground truth), while the red part represents the predicted brain tumor region. Outside blue areas represent normal brain regions, while outside red areas represent predicted normal brain regions.

To illustrate the calculation of the Dice score, let us consider the brain picture shown in Figure 3. The blue part of the image represents the real brain tumor region (ground truth), while the red part represents the predicted brain tumor region. Outside blue areas represent normal brain regions, while outside red areas represent predicted normal brain regions. When evaluating the performance of a medical image segmentation model, it is common to assume that positive samples represent brain tumors while negative samples represent normal brain tissue. The following metrics are used to evaluate the performance of the model:

True Positive (TP): This refers to the samples that are correctly identified as positive. In other words, the intersection of the blue and red area in the image represents true positive samples. True Negative (TN): This refers to the samples that are correctly identified as negative. In the image, the area other than the red and blue areas represents true negative samples. False Positive (FP): This refers to the samples that are incorrectly identified as positive but are actually negative. In the image, the red area except the blue area represents false positive samples. False Negative (FN): This refers to the samples that are incorrectly identified as negative but are actually positive. In the image, the part of the blue area except the red area represents false negative samples.

## 3 Results

### 3.1 Algorithm principle

#### 3.1.1 Network model

To improve the accuracy of segmentation of smaller regions of brain tumors (tumor core and enhanced tumor), this paper proposes a network model called MMGan based on a generative adversarial model. The proposed model comprises generative and adversarial model that work together to deliver superior segmentation results. The generative model consists of the encoding and decoding structure of the deep residual learning unit, with an attention mechanism added to the coding structure to extract shallow features from the deep residual structure. These features are then filtered by the attention mechanism and fused with the deep features of the decoding structure to enhance the segmentation accuracy. The adversarial network uses a convolutional neural network to discriminate between the segmentation results generated by the generative model and the expert segmentation results. By calculating the loss of the adversarial model, the generative model can be continuously optimized to achieve the optimal state of brain tumor segmentation.

**Figure 4:**
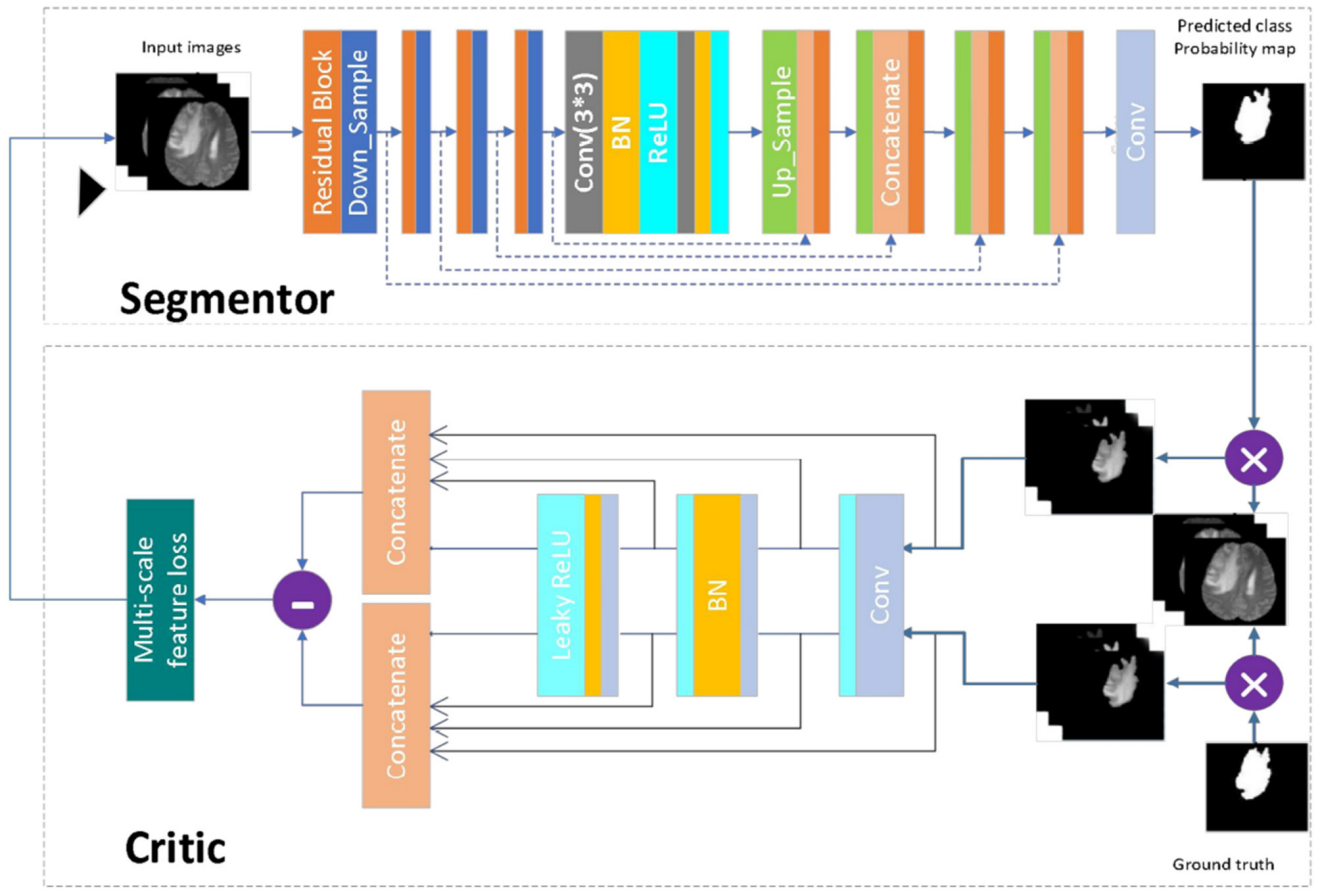
Network model diagram. First, the image to be segmented is input into the segmenter S, and the segmented result is output. The output segmentation result and the expert segmentation result are multiplied by the original image and input to discriminator D. The discriminator predicts the difference between the input results and transfers the loss to the generative model. After several iterations of confrontation, the discriminant loss tends to be zero. At this point, the generative model has reached the state of the optimal segmentation effect.

When the generative adversarial network performs the segmentation task, the generative model serves as the segmentation network, while the adversarial network judges the difference between the segmentation result of the segmentation network and the expert segmentation result. The loss is then calculated and used to optimize the gradient for the segmentation network to re-segment. Through the mutual iteration of the generative and adversarial model, the segmentation accuracy of the segmentation network is continuously improved. Figure 4 presents a schematic diagram of the model. First, the image to be segmented is input into the segmenter S, and the segmented result is output. The output segmentation result and the expert segmentation result are multiplied by the original image and input to discriminator D. The discriminator predicts the difference between the input results and transfers the loss to the generative model. After several iterations of confrontation, the discriminant loss tends to be zero. At this point, the generative model has reached the state of the optimal segmentation effect.

#### 3.1.2 Segmentation model

The segmentation model in this paper has been designed following the principle of semantic segmentation, as shown in Figure 4 (Segmentor). The residual network and the skip-connected encoder-decoder structure have been combined to achieve this. The generator is constructed using a 9-layer structure consisting of encoding, bridging and decoding parts. In the first section, the input image is encoded into a compact representation, while the last part restores the representation to pixel classification, i.e., semantic segmentation. The middle part serves as a bridge, connecting the encoding and decoding paths. All three parts comprise residual units consisting of two 3 × 3 convolutional blocks and a unit map. Each convolutional block comprises convolutional layers, BN layers and ReLU activation layers, with input and output linked by the identity map.

The encoding path consists of four residual units, with a pooling operation used in each unit to downsample the feature map size, reducing the feature map by half. Correspondingly, the decoding path also comprises four residual units, with feature maps upsampled from lower levels and concatenated with feature maps from the corresponding encoding paths before each unit. A 1 × 1 convolution is used to project the multichannel feature map into the desired segmentation after the last stage of the decoding path. Overall, there are 19 convolutional layers in total, and the parameters and output sizes of each step are shown in Table 1.

**Table 1:** Model parameters of the segmenter.

	**Unit level**	**Conv layer**	**Filter**	**Stride**	**Output size**
Input					160*160*4
Encoding	Level1	Conv1	3*3/4,64	1	
		Conv2	3*3/64,64	1	80*80*64
	Level2	Conv3	3*3/64,128	1	
		Conv4	3*3/128,128	1	40*40*128
	Level3	Conv5	3*3/128,256	1	
		Conv6	3*3/256,256	1	20*20*256
	Level4	Conv7	3*3/256,512	1	
		Conv8	3*3/512,512	1	10*10*512
Bridge	Level5	Conv9	3*3/512,1024	1	
		Conv10	3*3/1024,1024	1	10*10*1024
Decoding	Level6	Conv11	3*3/1536,512	1	
		Conv12	3*3/512,512	1	20*20*512
	Level7	Conv13	3*3/768,256	1	
		Conv14	3*3/256,256	1	40*40*256
	Level8	Conv15	3*3/384,128	1	
		Conv16	3*3/128,128	1	80*80*128
	Level9	Conv17	3*3/192,64	1	
		Conv18	3*3/64,64	1	160*160*64
Output		Conv19	1*1/64,3	1	160*160*3

#### 3.1.3 Discriminant model

The discriminant model as shown in Figure 4 (critic), uses an ordinary neural network to process the pre-segmentation and expert segmentation result, which are multiplied with the original image to form the model's input. The model then employs the multiscale L1 loss function to determine the difference between the two inputs, providing critical feedback for optimizing the segmentation model.

### 3.2 Loss function

This paper adopts the method of calculating the loss proposed in Jesson and Arbel (2018). Assuming that the dataset has *N* original training images *x*_*n*_ and real images *y*_*n*_, the multiscale target loss function is defined as follows:


(2) $$\begin{array}{l} \begin{array}{c} min\\ \theta_{S} \end{array}\begin{array}{c} max\\ \theta_{C} \end{array}L\left(\theta_{S},\theta_{C}\right)=\frac{1}{N}\sum_{n=1}^{N}l_{mae}\left(f_{C}\left(x_{n}{}^{\circ}S\left(x_{n}\right)\right),f_{C}\left(x_{n}{}^{\circ}y_{n}\right)\right)\\ \;=\frac{1}{NL}\sum_{N=1}^{N}\sum_{i=1}^{L}∥f_{C}^{i}(x)-f_{C}^{i}\left(\overset{´}{x}\right){∥}_{1} \end{array}$$

where θ_*S*_ and θ_*C*_ are the parameters of the segmenter and discriminator, respectively, L is the total number of layers in the discriminator network, and $f_{c}^{i}\left(x\right)$ represents the feature mapping of image x extracted at the ith layer of the discriminator.

By using a multiscale feature loss, the proposed method forces the segmenter and discriminator networks to learn hierarchical features of long and short spatial relationships between pixels.

### 3.3 Data preprocessing

**Figure 5:**
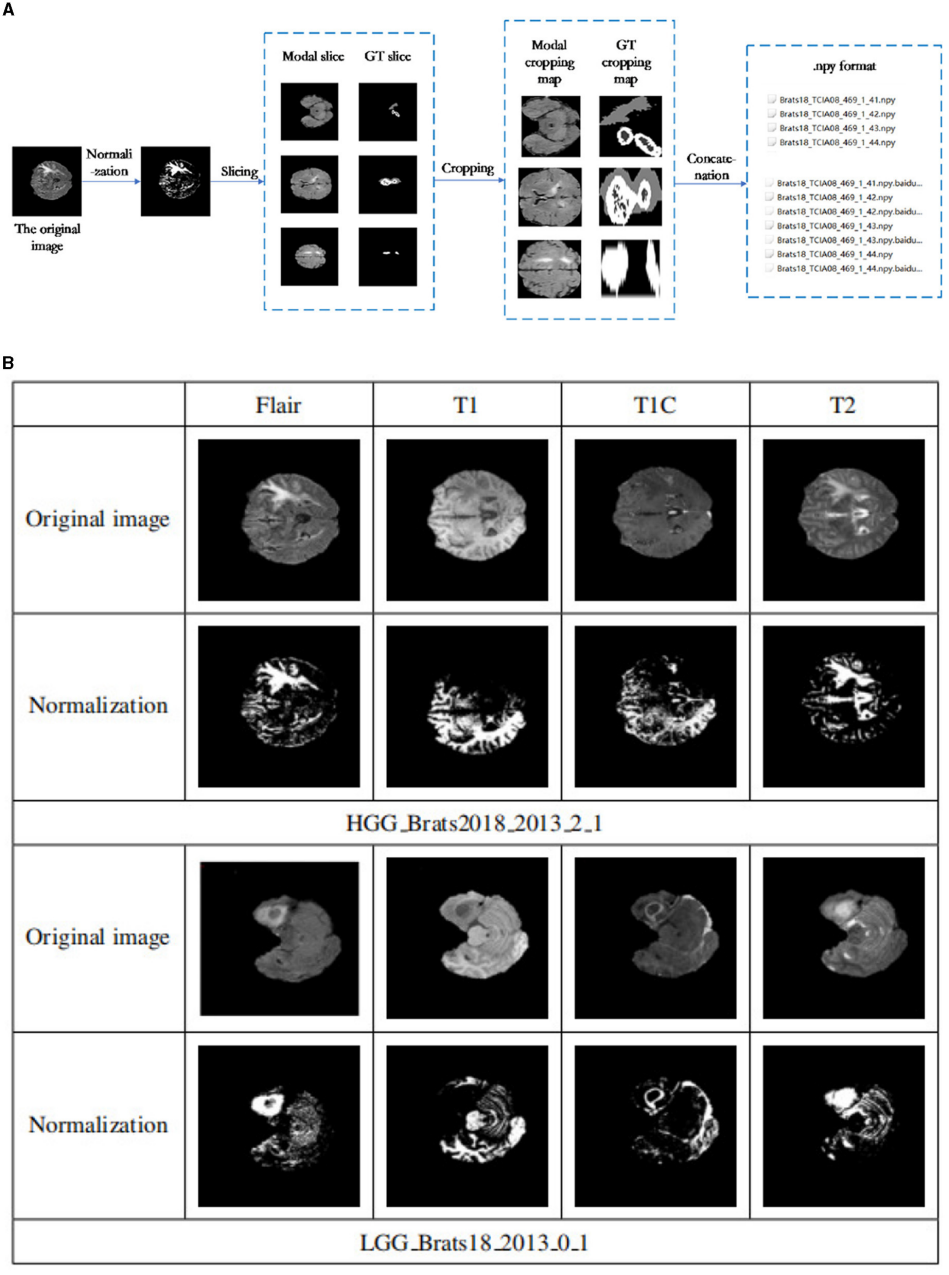
**(A)** Data preprocessing flowchart. The data preprocessing process was divided into four essential steps: normalization, slicing, cropping, and concatenation. **(B)** Data normalization results. The first and second rows are some examples of the four patterns of the Brats18 2013 2 1 sample in HGG and their corresponding normalized results. Similarly, examples of the four patterns of the Brats18 2013 0 1 sample in LGG and their corresponding normalized results are in the bottom two rows.

This experiment utilized the public BraTS 2018 and BraTS 2019 datasets to evaluate the proposed segmentation model's performance. The BraTS 2018 dataset was divided into 8:2 ratio for model training and validation, while an additional 50 cases from the BraTS 2019 data were used for model testing. Figure 5A shows that the data preprocessing process was divided into four essential steps: normalization, slicing, cropping and concatenation.

(1) Normalize each mode

To ensure consistency and remove the effects of anisotropy, the Z score was normalized for each of the four brain modalities used in this study (T1, FLAIR, T1c, and T2). This normalization process resulted in the mean and unit variance of the output images are zero. This is done by subtracting the mean and dividing by the standard deviation to count only brain regions.

Figure 5B shows the results before and after normalization of the dataset, where the first and second rows are some examples of the four patterns of the Brats18_2013_2_1 sample in HGG and their corresponding normalized results. Similarly, examples of the four patterns of the Brats18_2013_0_1 sample in LGG and their corresponding normalized results are in the bottom two rows. As show in Figure 5B, the image contrast of the four modes remains the same after data normalization.

The tumor area in medical brain images occupies only a small proportion, leading to a severe data imbalance problem. To address this issue, the level set method was employed to crop the image and improve the performance of the model segmentation. This method combines the *E*^*CA*^ energy functional of the Canny operator and the LBF energy function of the traditional level set to strengthen the detection of target edges while processing uneven grayscale images. This method accurately removes redundant background areas, effectively preventing multisegmentation of the target area and reducing the data imbalance issue.

(2) Slicing

**Figure 6:**
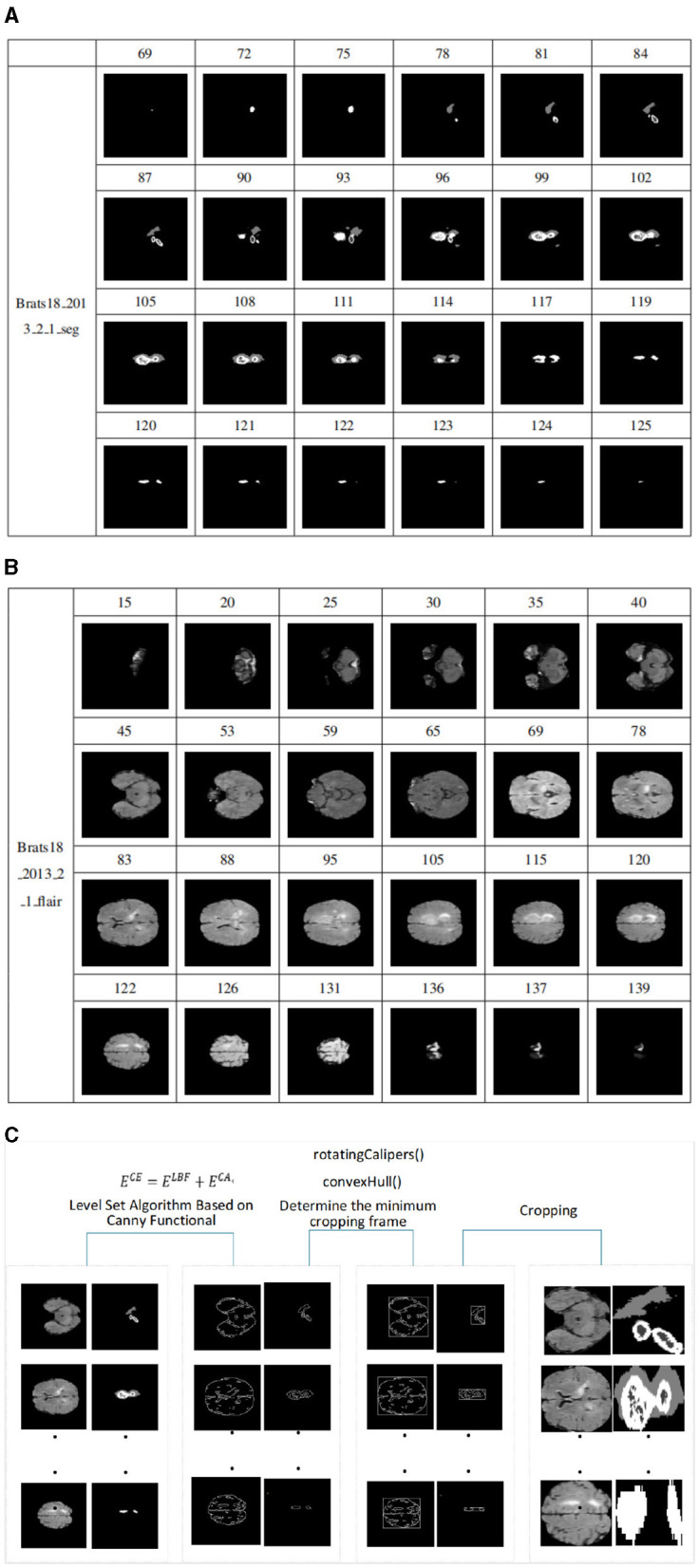
**(A)** Schematic diagram of size change of tumor in slices, sample from Brats18_2013_2_1_seg. It is the process of tumor growth from small to large and from large to small in the slices of sample Brats18_2013_2_1. **(B)** Schematic diagram of FLAIR image slice. It is the process of an image from large to small and from small to large in the FLAIR mode image slice of the sample Brats18_2013_2_1. **(C)** Model diagram of level set algorithm based on Canny functional.

Since most medical images are 3D data, they must first be converted into 2D data in order to be adaptable to 2D networks. Based on the 2D network, the 3D volume is processed into a sequence of 2D slices, the input network is segmented layer by layer, and then the segmentation results of each slice are combined into a volume output. Because axial plane slices are clearer and better rendered, axial plane slices are selected in this article. However, we found a steady increase or decrease in tumor size and shape in serial sections. The tumor first appears at a small size in any possible location of the image slice. The tumor will then remain in the same position in the image in subsequent slices, but it will be larger. The tumor will then begin to decrease in size after reaching the maximum size until it disappears completely. As shown in Figures 6A, B. In addition slices that do not contain lesions can be discarded during the slicing process, which can alleviate the problem of class imbalance.

A data sample can be cut into 155 slices. Figure 6A shows the process of tumor growth from small to large and from large to small in the slices of sample Brats18_2013_2_1. The slice number starts from 69, and every few slices are removed, for a total of 24 slices. Figure 6B shows the process of an image from large to small and from small to large in the FLAIR mode image slice of the sample Brats18_2013_2_1. Slice numbering starts at 15 and takes approximately every five slices for a total of 24 slices. As shown in Figures 6A, B, the size and distribution of images in each slice are different. Therefore, when cropping a slice, it is necessary to crop each image according to the size of the images in the slice.

(3) Level Set Algorithm Clipping Based on Canny Functional

In medical brain images, the gray area represents the brain region, while the black area represents the background. The background information accounts for a significant proportion of the entire image, making it challenging for image segmentation. Additionally, since the tumor region occupies only a small part of the whole image, so there is a severe data imbalance problem, with the black background area occupying a significant portion of the image. The data must be cropped to improve the model's segmentation performance. When a person looks at the image, the background information is automatically filtered out and all attention is focused on the brain area. Therefore, removing background information around brain regions is necessary for identification.

However, the sizes of the tumor and the brain in each slice image can vary significantly, making it challenging to use ordinary cropping methods. We employed the level set method to address this issue, which integrates the Canny operator into the traditional level set algorithm. The traditional level set algorithm has certain advantages in dealing with uneven grayscale images, and the integration of the Canny operator strengthens the detection of the target edge. This method accurately removes the redundant background areas, effectively preventing multisegmentation of the target area and reducing the data imbalance issue. The model diagram of this algorithm is shown in the Figure 6C, with the main modules being edge detection and determination of the cropping frame.

The level set algorithm is an algorithm for solving curve evolution problems. The principle is to treat the low-dimensional curve as the zero-level set of the high-dimensional surface and then segment the curve through the evolution iteration of the curve. The iteration time is shorter, and the result is more accurate when the initial contour contains more target regions.

The level set function is introduced into the energy function, and the LBF energy function is defined as follows:


(3) $$E^{LBF}(C,f_{1}(x),f_{2}(x))=\lambda_{1}\int_{\Omega }(\int_{outside(C)}K(x-y)|I(y)$$

(4) $$\begin{array}{l} -f_{1}(x){|}^{2}H_{\varepsilon }(\phi )\text{d}y)\text{d}x\\ +\lambda_{2}\int_{\Omega }\left(\int_{inside(C)}K(x-y)|I(y\right)\\ -f_{2}(x){|}^{2}(1-H_{\varepsilon }(\phi ))\text{d}y)\text{d}x \end{array}$$

where *C* is the closed initial contour curve, Ω is the image collection domain, and let λ_1_ = λ_2_ = 1. *K*(*x*) = *K*(|*x*|) is a non-negative monotone kernel function that depends on the gradient value of the image and has local properties. *f*_1_ and *f*_2_ are the grayscale fit values of the inner and outer regions of the image near point x. *H*_ε_(*x*) is the Heaviside function, and δ_ε_(*x*) is the derivative form of the Dirac function.

In medical image segmentation, it is essential to set appropriate threshold parameters to avoid inaccurate segmentation caused by too thick curves. Two threshold parameters, namely high and low, are required to accurately detect the edges of the target region. If the gradient of the point is greater than the high threshold, it is marked as a strong edge point. If the gradient is less than the low threshold, it is marked as a weak edge point. If the gradient falls between the high and low thresholds, the pixels in the eight fields surrounding the point must be analyzed. If there is a strong edge point in the vicinity, the point is marked as a strong edge point, and vice versa. Therefore, I(x, y) can be defined as a certain point in the image, and the Canny energy functional can be expressed as follows:


$$\begin{array}{l} E^{CA}=\iint_{\Omega }C(x,y)H(\phi )\text{dxdy}\\ \;=\iint_{\Omega }[\frac{G^{2}(x,y)}{x^{2}}+\frac{G^{2}(x,y)}{\partial y^{2}}]*I(x,y)H(\phi )\text{dxdy} \end{array}$$

where * is the convolution operation, *H*(ϕ) is the Heidegger function, and *G*(*x, y*) is the Gaussian kernel function, which solves the second-order partial derivatives for x and y, respectively.

Then, the total energy functional is:


(5) $$\begin{array}{l} E^{CE}=E^{LBF}+E^{CA} \end{array}$$

**Figure 7:**
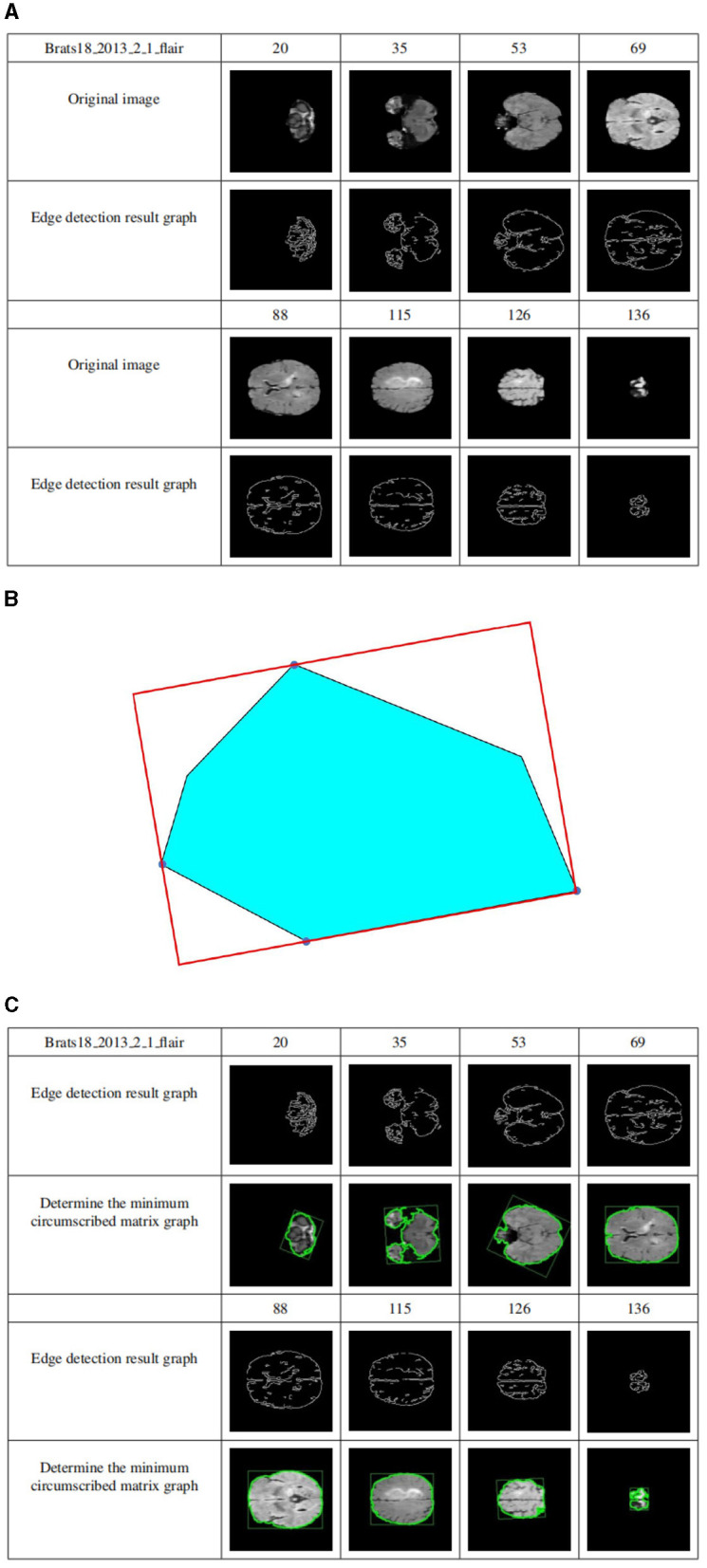
**(A)** Level set edge detection results based on Canny functional. The first row and the fourth row are the sample slice numbers, the second row is the different pictures after the sample is sliced, and the third row is the edge detection result of the slice image corresponding to the second row. The fifth and sixth rows are the same. **(B)** The circumscribed matrix of a convex polygon. The circumscribed rectangle is determined by four red tangent lines, one of which coincides with one side of the polygon. **(C)** Determine crop box results. The first row and the fourth row are the sample slice numbers, the second row is the sample edge detection result graph, and the third row is the result of determining the minimum external matrix corresponding to the second row. The fifth and sixth lines are the same.

Figure 7A shows the edge detection results of the sample Brats18_2013_2_1FLAIR mode based on the Canny functional level set. The first row and the fourth row are the sample slice numbers, the second row is the different pictures after the sample is sliced, and the third row is the edge detection result of the slice image corresponding to the second row. The fifth and sixth rows are the same. Figure 7A shows that the Canny operator can accurately detect the edges of brain tissue.

Compute the outer matrix of the minimum area of a 2D convex hull. The smallest circumscribed rectangle of a polygon must be collinear with one of its sides using geometry theorems. As shown in Figure 7B, the circumscribed rectangle is determined by four red tangent lines, one of which coincides with one side of the polygon.

Suppose the *n* vertices of the convex polygon P are entered in clockwise order. Calculate all 4 polygons, called Pxmin, Pxmax, Pymin, and Pymax, and construct 4 tangents through 4 points. They identified two “stuck” sets; if one or two lines coincided with one side, the area of the rectangle determined by the four lines was calculated and saved to the current minimum. Otherwise, the current minimum is defined as infinity. Rotate the lines clockwise until one of the lines coincides with one side of the polygon. Calculate the area of the new rectangle and compare it to the current minimum value. If it is less than the current minimum, it is updated, and the rectangle information is saved to determine the minimum value. Repeat the above steps until the line rotates at an angle greater than 90 degrees. Outputs the minimum area of the outer rectangle.

The cropping frame is determined by the above principles. Figure 7C shows the results of determining the minimum circumscribed matrix for data sample Brats18_2013_2_1FLAIR. The first row and the fourth row are the sample slice numbers, the second row is the sample edge detection result graph, and the third row is the result of determining the minimum external matrix corresponding to the second row. The fifth and sixth lines are the same. As shown in Figure 7C, regardless of how much the image occupies in the picture, their minimum circumscribed matrix can be accurately determined according to the detailed explanation of the Sklansky algorithm and rotation jamming algorithm.

**Figure 8:**
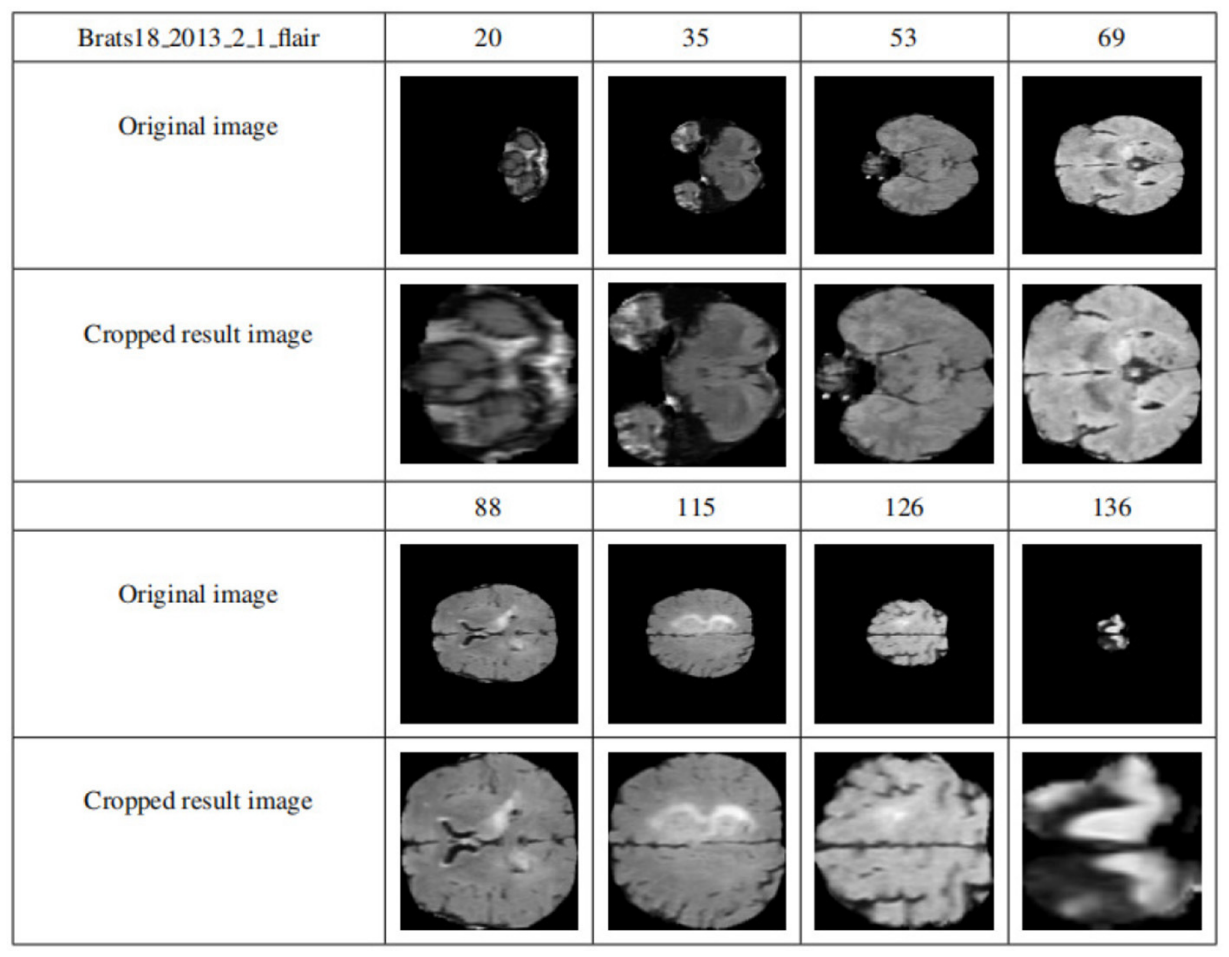
Display of results after data clipping. The first row and the fourth row are the sample slice numbers, the second row is the different slice images of the sample, and the third row is the cropped result image corresponding to the second row. The fifth and sixth rows are the same.

By applying the cropping frame, the black background is removed to the maximum extent, thereby reducing the data imbalance and making the subsequent brain tumor segmentation training more accurate. Figure 8 shows the comparison results of the data sample Brats18_2013_2_1FLAIR before and after cropping. The first row and the fourth row are the sample slice numbers, the second row is the different slice images of the sample, and the third row is the cropped result image corresponding to the second row. The fifth and sixth rows are the same. As shown in Figure 8, each slice has a black background cropped to the maximum extent without excessive cropping.

(4) Multimodal slice channel connection

Since slices are multimodal, the slices of each modality should be combined into multichannels and finally saved as NumPy, while the corresponding GT slices are saved directly as NumPy. The specific implementation is that each mode of each data sample has 155 slices, and one slice is taken from each mode according to the slice number and placed in a four-dimensional vector that is filled with 0. Specifically, the 0-dimension stores the FLAIR model modal slices, 1-dimensional storage of T1 modal slices, the 2-dimensional storage of T1c modal slices, and the 3-dimensional storage of T2 modal slices.

**Figure 9:**
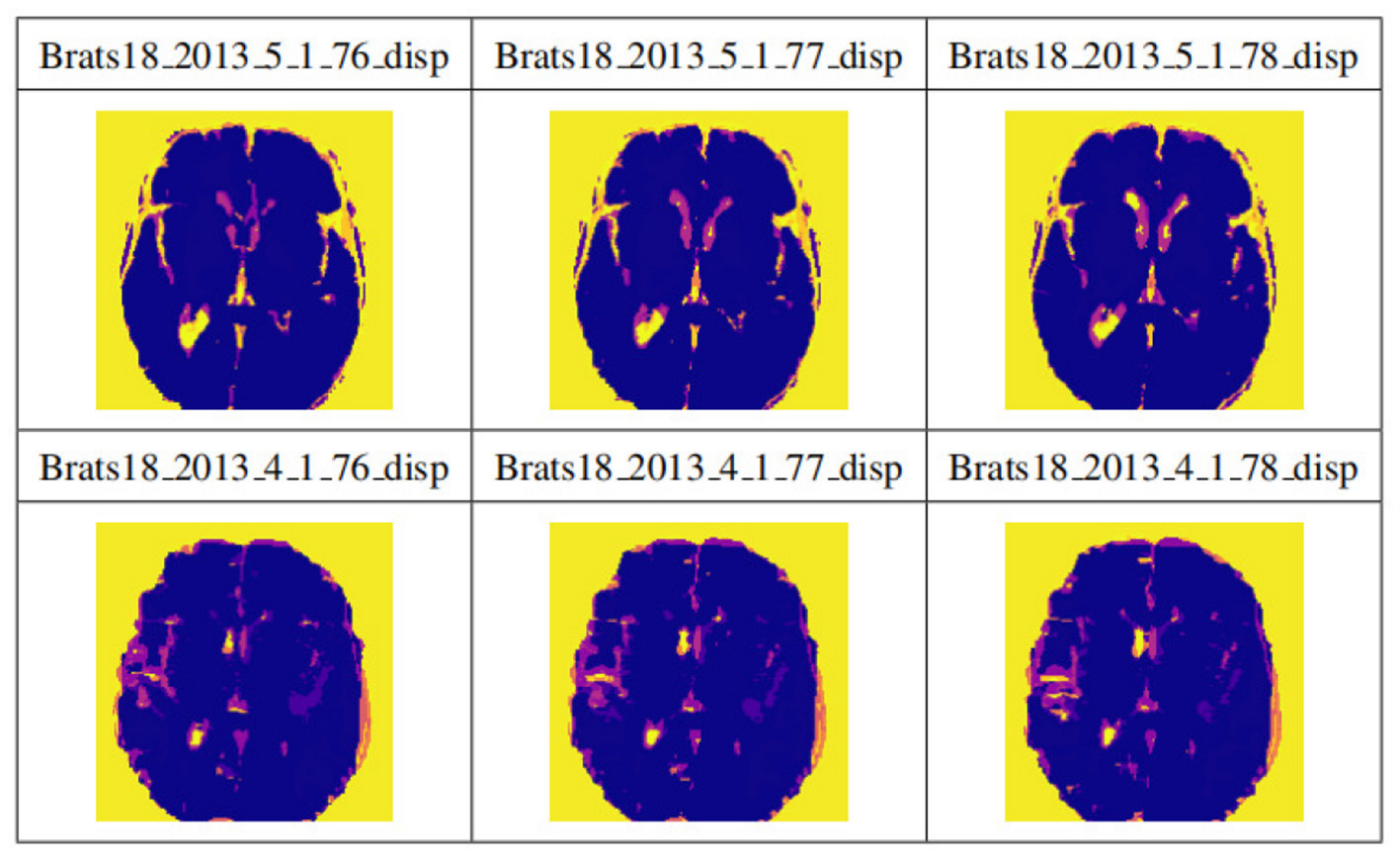
Display of data preprocessing results. The connection results of channels 76, 77, and 78 in the four modal slices of Brats18_2013_5_1 are listed in the second row, while the connection results of the four modal slice labels of Brats18_2013_4_1 are in the fourth row.

Figure 9 shows the visualization results after saving the data in .npy format. Two data samples are shown, Brats18_2013_5_1 and Brats18_2013_4_1. The connection results of channels 76, 77, and 78 in the four modal slices of Brats18_2013_5_1 are listed in the second row, while the connection results of the four modal slice labels of Brats18_2013_4_1 are in the fourth row.

### 3.4 Influence of the network model on the experimental results

**Figure 10:**
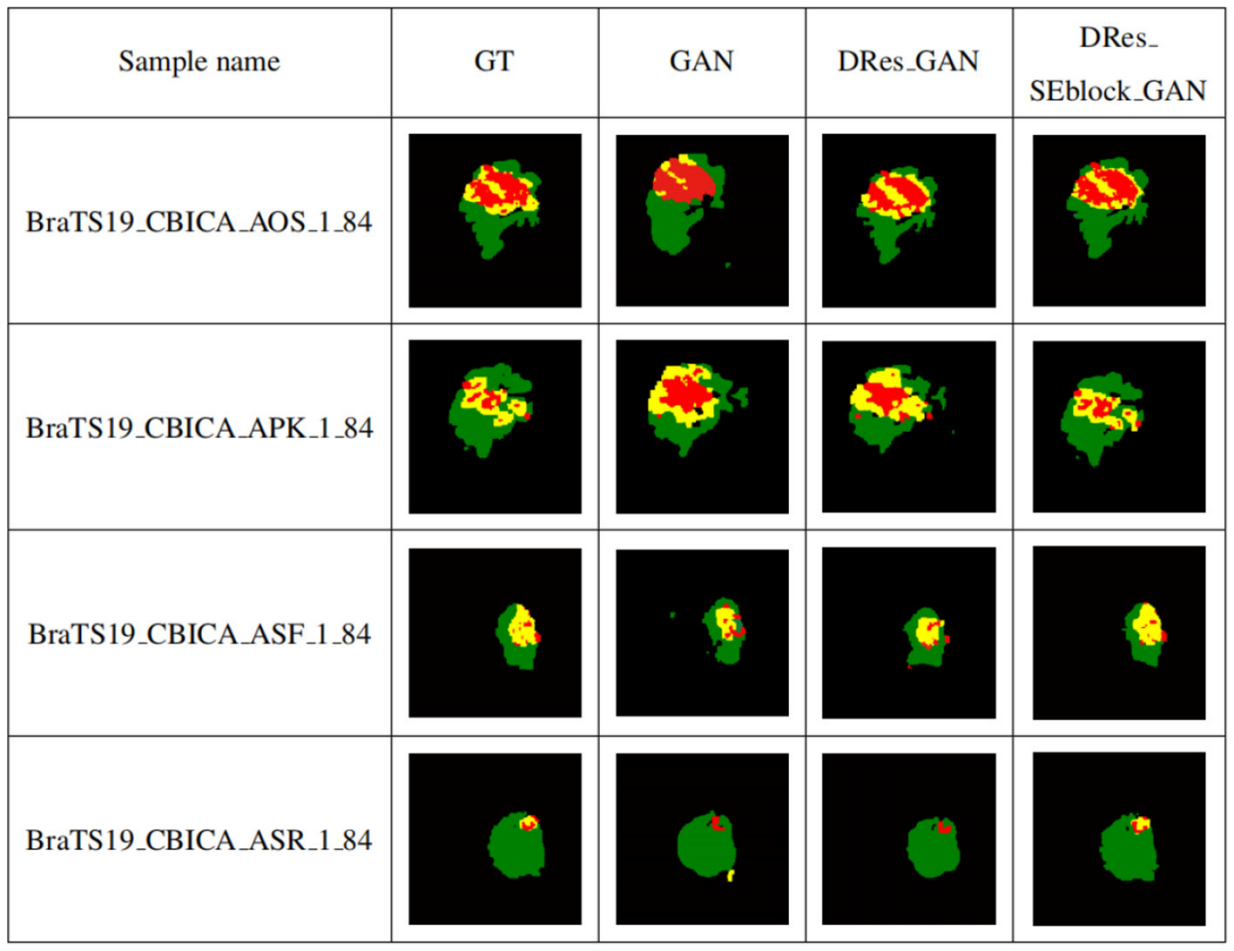
Self-contrasting experiment results. The first column shows the brain tumor segmentation results by experienced experts. The second column shows the results of the original generative adversarial network segmentation. The third column shows the results of the segmentation using the deep residual learning unit-based encoding structure. The fourth column shows the segmentation results of the proposed model, which incorporates a deep residual learning unit and an attention mechanism.

To verify the performance of the proposed model for medical brain image segmentation, it is compared with three other models, including the original model, the model with residual structure added, and the model with attention mechanism added. The ablation experiment results are shown in Figure 10, with four samples per row. The first column shows the brain tumor segmentation results by experienced experts. The second column shows the results of the original generative adversarial network segmentation. The third column shows the results of the segmentation using the deep residual learning unit-based encoding structure. The fourth column shows the segmentation results of the proposed model, which incorporates a deep residual learning unit and an attention mechanism.

As shown in Figure 10, GAN enhances some detailed features of brain tumors, but it is prone to over-segmentation and generates more isolated points. The segmentation results of the DRes_GAN model have a clear boundary, but the segmentation of the brain tumor boundary is not delicate enough. The proposed model in this paper overcomes the limitations of other algorithms and achieves similar results to expert segmentation, with high accuracy in extracting the boundary of brain tumors and effectively solving the problems of over-segmentation and under-segmentation.

Table 2 shows the quantitative evaluation results of adding different modules to the GAN network structure. To ensure the reliability of the experimental results, the ablation experiments train different networks under the same experimental parameters. Among them, GAN is the original generative adversarial network method, DRes_GAN is a method that substitutes a deep residual structure for the original GAN's generative model, and DRes_SEblock_GAN is a method that substitutes a deep residual structure combined with an attention mechanism which is the improvement method proposed in this paper.

**Table 2:** Ablation Experiment.

**Method**	**WT**	**TC**	**ET**	**Average**
GAN	0.81	0.70	0.66	0.72
DRes_GAN	0.83	0.81	0.77	0.80
DRes_SEblock_GAN	**0.87**	**0.86**	**0.78**	**0.84**

GAN is the original generative adversarial network method, DRes_GAN is a method that substitutes a deep residual structure for the original GAN's generative model, and DRes_SEblock_GAN is a method that substitutes a deep residual structure combined with an attention mechanism which is the improvement method proposed in this paper. Bold indicates optimal results and underlined parts indicate sub-optimal results.

It can be seen from the Table 2 that with the addition of modules, the complexity of the network structure increases, which verifies the role of the deep residual learning unit and the attention mechanism. By adding a deep residual block, the segmentation accuracy for enhanced tumor regions and tumor core regions is improved by 11%, and the segmentation performance for whole tumor regions is also improved. The addition of the attention mechanism further improves the segmentation accuracy of different tumor regions.

### 3.5 Comparing the experimental results of different algorithms

**Figure 11:**
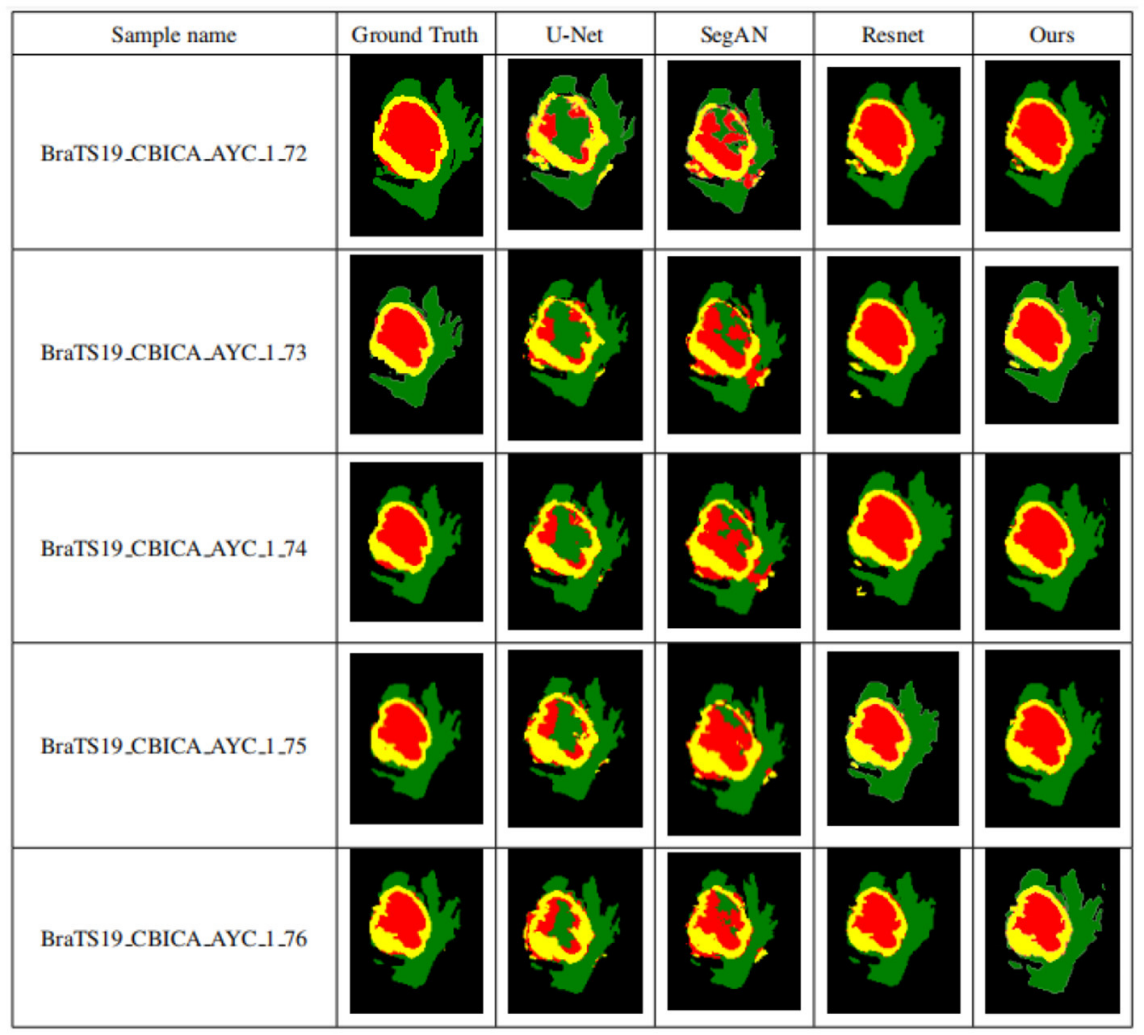
Segmentation results of different models. The first column shows the expert segmentation results, the second column shows the U-Net model segmentation results, the third column shows the SegAN model segmentation results, the fourth column shows the ResNet model segmentation results, and the fifth column shows the segmentation results of the proposed generative adversarial network.

To validate the performance of the proposed brain tumor segmentation model, it is compared with other state-of-the-art models, including the U-Net model proposed in Zheng et al. (2021), the ResNet model proposed in Chen et al. (2022), and the SegAN model proposed in Xue et al. (2018). Figure 11 presents the segmentation results of each model, with the first column showing the expert segmentation results, the second column showing the U-Net model segmentation results, the third column showing the SegAN model segmentation results, the fourth column showing the ResNet model segmentation results, and the fifth column showing the segmentation results of the proposed generative adversarial network.

As seen from Figure 11, the other three models have obvious differences in the segmentation of smaller areas of brain tumors, such as the enhanced tumor and tumor core, when compared to the expert segmentation results in the first column. The area segmented by other models is smaller than that segmented by the expert, and there are small spots of different colors in the tumor core area, indicating that the smaller area of the brain tumor is under-segmented. The proposed algorithm in this paper achieves segmentation results similar to those of experts with high accuracy compared to other methods. Higher segmentation accuracy can be obtained for smaller tumor areas, which solves the problem of under-segmentation of small areas by other algorithms.

## 4 Discussion

GANs have made significant breakthroughs in various fields, inculding image classification, object detection, and high-resolution image generation. In medical image segmentation, GANs have made the segmentation results more continuous, effectively addressing the issue of image segmentation results being quite different from the gold standard. Adversarial networks were first applied to image segmentation tasks by Dey and Ashour (2018). Since then, many studies have applied GAN-based methods to efficient brain tumor segmentation (Dey and Ashour, 2018; Jain et al., 2021; Rao and Karunakara, 2021). In these research works, Luc et al. (2016) proposed the application of GANs to image semantic segmentation, where the discriminator needs to judge the difference between the real segmentation and the actual segmentation mask. Experimental results have demonstrated that this method reduces the possibility of overfitting. However, a significant disadvantage of the original GAN is that the loss function of the discriminator produces a single integer. The discriminator either reports whether the input segmentation masks are raw or generated or informs whether the input samples are from the same domain as the source database. The discriminator loss function's gradient flow is insufficient for feature learning in the segmenter network for a single scalar or Boolean output. Xue et al. (2018) proposed an adversarial network with a multiscale L1 loss function called SegAN. The discriminator in SegAN extracts hierarchical features from the input image, the discriminative network aims to maximize the mean absolute error (MAE) or L1 distance between two hierarchical features, and the segmenter generates a segmentation mask with the slightest error. While SegAN can extract features at different levels, segmentation of relatively small regions such as core and enhancing tumors may require more attention to pixel-level features. Mondal et al. (2018) applied GANs for the first time to semi-supervised segmentation of 3D multimodal brain images and the segmentation performance was significantly improved compared to fully supervised methods. Zhao et al. (2018) achieved accurate segmentation of craniomaxillofacial (CMF) bone structures in MRI and CT images. Deng et al. (2022) used a residual structure and attention mechanism to segment the hippocampus in a generative adversarial network. Oh et al. (2020) used the CGAN and pix2pix framework to segment white matter in 18F-FDGPET/CT images, achieving high-precision brain tissue segmentation. Gu et al. (2019) and Li et al. (2019) used GAN to segment neonatal brain images to aid in neonatal disease diagnosis. MarcoConte et al. (2021) used GAN to synthesize missing MRI sequences and demonstrated that GAN-generated images can effectively assist other deep learning models for segmentation. To further improve the accuracy of brain tumor segmentation and the quality of manually segmented labels, Cheng et al. (2021) used GAN to perform label correction on samples.

The transformer based on the encoder-decoder structure has demonstrated remarkable efficiency in computer vision tasks such as image classification, recognition, and segmentation (Liu et al., 2023). Chen et al. (2021) used transformers as strong encoders combined with U-Net to recover local spatial information to enhance finer details of medical image segmentation tasks. In multiorgan Segmentation and cardiac segmentation, TransUNet has surpassed other competing methods. Liu et al. (2022b) addressed the resolution gap between training instability in large-field view model training and application, pretraining and fine-tuning, and hunger for labeled data. The residual postnorm method combined with cosine attention was used to improve the training stability. A continuous position biasing method with logarithmic intervals is used to effectively migrate models pretrained with low-resolution images to downstream tasks with high-resolution inputs. SimMIM, a self-supervised pretraining method, reduces the need for massive labeled images. Shen et al. (2023) designed boundary-guided transformers to accurately separate rectums from tumors, outperforming 6-year-experienced surgeons (*p* < 0.001). Wang et al. (2021) used the Transformer in 3D CNN for the first time for MRI brain tumor segmentation, inheriting the advantages of 3D CNN modeling local context information and using Transformer to learn global semantic correlation. Cao et al. (2023a) proposed Swin-Unet, a class U-Net pure Transformer for medical image segmentation, using a Transformer-based U-type encoder-decoder architecture with hopping connections for local global semantic feature learning. Liu et al. (2022a) introduced multimodal image fusion techniques for brain tumor segmentation, including pixel and feature-level fusion. Cao et al. (2023b) proposed a 3D convolutional neural network MBANet with 3D multi-branch attention, significantly improving performance compared to other state-of-the-art methods. Cheng et al. (2020) proposed an improved multitask learning method that uses a lightweight network with only two scales to segment different types of tumor regions, achieving high Dice coefficients in the WT region segmentation.

Experiments show that adding a deep residual learning unit to the generative adversarial network, deepening the number of network layers, and reducing the network parameters greatly improve the segmentation performance of the network. At the same time, adding attention modules and distributing weights to convolutional layers of different sizes effectively promotes the utilization of features at different levels. Experiments show that the network model proposed in this paper can promote the accuracy of segmentation and effectively and accurately complete the task of segmenting brain tumors.

Table 3 summarizes the comparison of Dice scores between the proposed method and several state-of-the-art segmentation methods, including the U-Net model proposed in Zheng et al. (2021), the ResNet model proposed in Chen et al. (2022), and the SegAN model proposed in Xue et al. (2018), the TransBTS model proposed in Wang et al. (2021), the Swin-Unet model proposed in Cao et al. (2023a), the PFFFNet model proposed in Liu et al. (2022a), the MBANet model proposed in Cao et al. (2023b), and the PGMLNet model proposed in Cheng et al. (2020). These dice scores range from 0.80 to 0.90 for whole tumor segmentation using these improved segmentation methods. The proposed method achieves the highest dice scores for tumor core and enhanced tumor region segmentation, with scores of 0.86 and 0.78, respectively, while ensuring a high dice score for total tumor segmentation. Furthermore, our model achieves the highest average value when the averages for the whole tumor, tumor core, and enhanced tumor dice scores are considered. These results clearly demonstrate that the improved method in this paper outperforms other algorithms.

**Table 3:** Dice comparison of different segmentation methods.

**Method**	**WT**	**TC**	**ET**	**Average**
U-Net (Zheng et al., 2021)	0.80	0.63	0.60	0.68
SegAN (Xue et al., 2018)	0.85	0.70	0.66	0.74
Resnet (Chen et al., 2022)	0.87	0.74	0.77	0.79
TransBTS (Wang et al., 2021)	0.88	0.81	**0.78**	0.82
Swin-Unet (Cao et al., 2023a)	0.89	0.78	**0.78**	0.82
PFFFNet (Liu et al., 2022a)	0.89	0.81	0.77	0.82
MBANet (Cao et al., 2023b)	0.89	0.83	**0.78**	0.83
PGMLNet (Cheng et al., 2020)	**0.90**	0.82	0.76	0.83
MMGan (ours)	0.87	**0.86**	**0.78**	**0.84**

The WT, TC, and ET regions as well as the average of the three regions are calculated and displayed, where the optimal results are indicated in bold font and the suboptimal results are underlined.

We further investigated the statistical significance of the performance improvement for the proposed MMGAN using the paired *t*-test. The *p*-values are listed in Table 4, respectively. As shown in Table 4, compared with the other supervised learning-based methods, the proposed MMGAN achieved significant improvement in terms of the main evaluation metrics (Dice), with *p*-values less than 0.05. Table 4 further proved the effectiveness of the proposed MMGAN. Compared with those of the other methods, the segmentation accuracies of both MMGAN have been significantly improved.

**Table 4:** The statistical significance of the performance improvement for the proposed MMGAN using the paired *t*-test.

	***P*-value**
MMGan - U-Net (Zheng et al., 2021)	< 0.001
MMGan - SegAN (Xue et al., 2018)	0.008
MMGan - Resnet (Chen et al., 2022)	0.003
MMGan - TransBTS (Wang et al., 2021)	0.009
MMGan - Swin-Unet (Cao et al., 2023a)	0.032
MMGan - PFFFNet (Liu et al., 2022a)	0.015
MMGan - MBANet (Cao et al., 2023b)	0.020
MMGan - PGMLNet (Cheng et al., 2020)	0.038

In this study, for the brain tumor data preprocessing and segmentation method, the accuracy of brain tumor segmentation is improved by improving the network model, and some results are proved by experiments. However, There are some limitations of this work. Firstly, we only improve the segmentation model with the structure of the segmentation model by combining the deep residual structure and the attention mechanism. Subsequently, the hyperparameters of the model can be adjusted by correcting the discriminative network to achieve better training results. Secondly, we only use the multi-scale L1Loss function to compute the loss to optimize the network, in recent years, there are many methods that have been proposed to compute the loss, such as the common cross-entropy Loss function (Yeung et al., 2022), the absolute value Loss function, the square Loss function and so on for comparison. Therefore, in the subsequent research, different loss functions can be applied to compute the loss to optimize the network and compare the differences between them and their impact on the network performance. Finally, designing and training deep neural networks from scratch for a specific task can be a daunting and time-consuming process. Different structures and initialization procedures can significantly impact the final performance of the neural network. However, transfer learning can be viewed as a solution for transferring information gathered in the source domain and then fine-tuning it in the target domain to obtain satisfactory performance, assuming that the distance between the target and source domains is close enough. Using pre-trained models from state-of-the-art algorithms is a common transfer learning scheme, providing an efficient way for brain tumor segmentation using information from brain tissue segmentation or abnormal segmentation of other organs.

## 5 Conclusion

Segmenting lesions or deformities in medical images requires greater precision than in natural images. Small segmentation errors in medical images can mislead inexperienced users or significantly affect computer-aided care. Therefore, in medical image segmentation, it is necessary to develop a model that can accurately restore the details of the target object. In this paper, several research experiments were conducted to achieve accurate segmentation of multimodal brain tumor data. These experiments included using the *E*^*CA*^ energy function of the Canny operator combined with the LBF energy function of the traditional horizontal set to strengthen the detection of the target edge, cropping each modal image to more accurately remove the redundant background area, adopting the U-shaped codec structure in the generative model, and using the deep residual unit as the basic structure of the network to improve the problem of training difficulties faced by deep learning networks. These improvements significantly improved both the gradient vanishing of the training process and the strength of its correlation. To further improve the accuracy of the segmentation of different regions of brain tumors, attention mechanisms were added during the coding stage to add a signal that is more sensitive to smaller details. Finally, qualitative and quantitative experiment analysis proved the improved model's correctness. Subsequently, the discriminant network can be modified to adjust the model's hyperparameters to achieve better training results. Different loss functions can also be applied to calculate losses to optimize the network, such as the common cross-entropy loss function, absolute value loss function, square loss function, etc., and compare these different loss functions and their impact on network performance.

## Data availability statement

The original contributions presented in the study are included in the article/supplementary material, further inquiries can be directed to the corresponding author.

## Author contributions

LG: Formal analysis, Validation, Writing – original draft, Writing – review & editing. JL: Formal analysis, Writing – original draft. RZ: Data curation, Validation, Writing – original draft. HB: Writing – original draft. JW: Writing – original draft. YC: Data curation, Validation, Formal analysis, Writing – original draft. HD: Funding acquisition, Project administration, Supervision, Formal analysis, Writing – original draft.
